# Murals meet microbes: at the crossroads of microbiology and cultural heritage

**DOI:** 10.15698/mic2021.12.765

**Published:** 2021-12-06

**Authors:** Maria A. Bauer, Katharina Kainz, Christoph Ruckenstuhl, Frank Madeo, Didac Carmona-Gutierrez

**Affiliations:** 1 Institute of Molecular Biosciences, NAWI Graz, University of Graz, Graz, Austria.; 2 BioHealth Graz, Graz, Austria.; 3 BioTechMed Graz, Graz 8010, Austria.

**Keywords:** biocleaning, tangible cultural heritage, microbial deterioration, biodeterioration, biotreatment, bacteria, fungi, biofilm

## Abstract

Our cultural heritage consists of manifold cultural expressions and represents a defining feature of our societies that needs to be further inherited to future generations. Even though humankind always fought a daily struggle for survival, at the same time, it seemed to have a spiritual need that went far beyond mere materialistic satisfaction and nowadays manifests in sometimes very ancient, yet brilliant artistic works. This fundamental legacy is endangered by several instances, including biodeterioration. Indeed, microorganisms play a significant role in the decline of all forms of tangible cultural heritage, including movable, immovable and underwater cultural heritage. Microbial colonization, biofilm formation and damaging metabolite production eventually result in critical decay. Thus, efforts to mitigate the negative impact of damaging microorganisms have been pursued with diverse physical, chemical and biological approaches. Intriguingly, recent advances have unveiled that specific microorganisms and microbial-based technologies also have the potential for cultural heritage preservation and present unique advantages. This short piece provides a quick overview on the duality of microorganisms in the conservation and restoration of cultural heritage.

“He who cannot be far-sighted, Nor three thousand years assay, Inexperienced stays benighted, Let him live from day to day.” is a famous quotation from Goethe (rendered to English by J. Whaley) [1]. In other words: somebody is not aware what happened in the last three thousand years, such person remains ignorant, merely surviving from day-to-day. The interest to preserve and inherit cultural value for future generations has accompanied humankind since ancient times. However, it has been constantly and severely challenged not only by the ravages of time, but also by wars, colonial conquests and imperial invasions. The legal grounds to protect this heritage were set in the 19^th^ century and were extended in the 20^th^ century, among others by organizations like the international body UNESCO. Nowadays, it is understood that cultural heritage (CH) refers to manifold aspects with implications on multiple levels. It can be defined as “an expression of the ways of living developed by a community and passed on from generation to generation, including customs, practices, places, objects, artistic expressions, and values.” [2]. Accordingly, CH refers not only to tangible culture, *inter alia* buildings, wrecks, landscapes, books, or art, but also to intangible culture, which includes *e.g.*, traditions, performing arts, rituals, knowledge as well as skills and practices to produce tangible culture or traditional crafts. Both tangible and intangible CH need to be safeguarded with active effort so that future generations can enjoy, benefit and learn from the past. CH not only can evoke certain emotions and bring countries and communities together, it can also be of economic value as a driver of sustainable development and local job creation. Thus, to successfully achieve CH preservation, the work of different disciplines, including conservator scientists, historians, chemists, microbiologists and many others needs to be connected.

An effective way to preserve intangible CH is to raise awareness for it and share it with others, i.e. “it must remain relevant to a culture and be regularly practiced and learned within communities and between generations” [3]. The preservation of tangible CH poses many different challenges with tangible CH including movable (paintings, sculptures, coins, manuscripts), immovable (monuments, archaeological sites, etc.) and underwater (shipwrecks, underwater ruins and cities) items. Accordingly, tangible CH comes in the shape of a myriad of different objects, composed of a large range of diverse materials, among others, stone, wood, metal, paper, ceramics, synthetic materials, or organic matter. Additionally, tangible CH can exist either in controlled and monitored indoor environments, but also in outdoor and under largely uncontrolled conditions. Therefore, all tangible CH is subject to physical, chemical and/or biological deterioration. Thereby, biodeterioration, and more specifically the impact of microbial biodeteriogenic agents (such as bacteria, algae, lichens, archaea, fungi) represent a central risk for CH [4].

Microbial deterioration defines any unwanted modification in the characteristics of a given material by the vital activity of microbial organisms [5]. Ultimately, it represents the consequence of microorganisms ensuring their survival in different environments through degradation pathways used for nutrition, morphological adaptations (biofilms) and the generation of metabolic products, which may include acids, solvents, surfactants, or pigments. All may contribute to the deterioration of CH, ranging from slight aesthetic alterations to severe physical and chemical damages [6]. If microbial colonization does have negative consequences for the integrity of CH, steps have to be taken to mitigate the effect.

Accordingly, many efforts have been directed towards both identifying damaging microorganisms and neutralizing their impact on the deterioration of any given item of endangered tangible CH. Regarding microbial identification, three main approaches can be distinguished [7]. (i) Culture-independent meta-omic analyses, in which the whole microbial community is analyzed using, among others, metagenomics, metatranscriptomics, metabolomics, metaproteomics, PCR amplicons and shotgun sequencing. (ii) Culture-dependent analyses of isolates, which is the most commonly used tool to identify microorganisms on CH and can include, for example, taxonomic classification (*inter alia* sequencing-based approaches) or miscellaneous activity tests under different conditions (pH, temperature, etc.) [8, 9]. This approach is simple, cheap, and can be accomplished, for instance, by museum workers with a basic training. However, many CH-resident microorganisms are unfortunately not cultivable. Therefore, a combined strategy with meta-omic analyses would provide the most reliable results. As a third identification approach, changes in the structure and composition of degraded or renovated material can be examined by (iii) physico-chemical analyses. These include, among others, scanning electron microscopy (coupled with energy-dispersive X-ray spectroscopy), transmission electron microscopy, fluorescence microscopy, Raman spectroscopy, Fourier transform coupled infrared spectroscopy, ion chromatography and microtomography. Some of these methods not only help monitoring the levels of microbial contamination, but may also reveal the actual state of damage of the analyzed artwork [7].

Upon recognition of the microbial threat, strategies for the removal of the identified microorganisms or for the neutralization of their negative effects need to be found. A plethora of traditional and novel methods and techniques are used, including physical methods (*e.g.*, gamma radiation and UV-C irradiation, low pressure water rinsing, steam cleaning, laser cleaning, heat-shocking, microwave and dry-ice treatment) as well as chemical treatments (traditional biocides and nanoparticles). Additionally, biological procedures have been developed, including biocidal treatments with natural compounds [6, 10] or, more recently, biocleaning methods, where enzymatic or organismal treatment is used for the removal of specific metabolites, substrates and/or other damaging material (see below) [11–19]. Unfortunately, conventional physico-chemical methods are not always effective and in some cases may even be dangerous for the treated CH. Some chemical treatments (nanoparticles, traditional chemical biocides) on stone CH, for instance, have been shown not to be sufficiently selective and consequently damage the stone surfaces [11, 20–23]. Other procedures need to be applied repeatedly (*e.g.*, mechanical removal, gamma radiation, UV-C irradiation) and may therefore damage stone surfaces as well as organic heritage material like wood, leather, parchment, and textiles [6]. In addition, some active compounds used for CH preservation can be toxic and not degradable, or can be persistent in and thus negatively impact the environment. Thus, it continues to be an important task to refine existent CH preservation methods and to further elucidate novel (and mild) treatments with high effectivity that neither pose a health risk nor compromise the environment.

While efforts to counteract damaging microbial activity on CH remain an important issue, microorganisms may themselves also be helpful for heritage conservation and restoration. In fact, the metabolism of many microorganisms may be deleterious on one substrate, but can be positively exploited on a different substrate. For example, while bacterial sulfate reduction accounts for biocorrosion of iron and iron alloys [24, 25], black crusts can be removed from stone artwork with the help of distinct microorganisms that use sulfate reduction pathways [14, 20]. In fact, biocleaning methods have been developed, where selected bacteria and fungi as well as microbial enzymes are used for the removal of damaging sulfates, nitrates, animal glue, caseine as well as organic matter from stone [11–16] or other surfaces [17–19, 26, 27]. Biocleaning is less invasive and an environmentally friendly alternative, since the microorganisms used act similarly on CH as in their natural environment. Examples include the bacteria *Pseudomonas denitrificans, which has been applied to eliminate nitrate salts* [28]*, Desulfovibrio vulgaris*, which have been used to remove sulfates [15]. or *Pseudomonas stutzeri* which can be employed for removal of organic matter [19]. Moreover, microbial treatment may be effective through other processes, such as biological patination of metals by *Beauveria bassiana* [29], or carbonatogenesis in self-healing concrete mediated by bacterial spores of *Bacillus alkalinitrilicus* [30]. Additionally, while microbial biofilms mainly do have deteriorating effects on CH, some biofilms, especially those generated by particular cyanobacteria and fungi, have been shown to conserve lithic materials by generating a stable layer that contributes to, for example, case-hardening of sandstone and protects the surfaces from further decomposition [31–33]. Moreover, natural biofilms can be more compatible with the surface of CH than traditionally used protective coatings [6].

Biocleaning is inexpensive, easy to use and has a low negative impact on CH, especially on stoneworks, mural paintings and frescoes, but also on paper, wood, waterlogged wood and iron [17]. Nevertheless, it presents a number of limitations. If water is applied on CH surfaces during biocleaning treatment, it may cause leaching or salt solubilization, and also may negatively affect the state of CH materials. To circumvent this problem, the so-called dry biocleaning approach has been devised, where dehydrated microbial cells could be employed without using free water or gel-based matrices. In this respect, the baker's yeast *Saccharomyces cerevisiae* has recently been successfully implemented on CH preservation [11]. Of note, dry biocleaning could be particularly efficient in removing alterations on highly ornamented stoneworks [11]. Still, the application of microorganisms – generally conceived as CH-deteriorating agents - continues to meet some reluctancy, which tends to favor the chemical and mechanical treatments [28]. Indeed, more work is needed to study the safety of biocleaning technology, in particular with respect to the CH objects themselves, but also regarding possible medical and environmental hazards [34]. Thus, the close monitorization of both short-term and long-term effects of bio-restored CH will help clarify whether this method is indeed an overall softer and safer process. This will also help decipher if microbial agents used for biocleaning need to be removed after usage at the CH site [17] or may remain under constant surveillance [34]. In any case, for the further use and acceptance of biocleaning strategies, it will be important to establish techniques and protocols to define if a given microorganism does cause biodeterioration, promotes only aesthetic alterations or, in fact, acts in a bioprotective manner [6].

Biocleaning is inexpensive, easy to use and has a low negative impact on CH, especially on stoneworks, mural paintings and frescoes, but also on paper, wood, waterlogged wood and iron [17]. Nevertheless, it presents a number of limitations. If water is applied on CH surfaces during biocleaning treatment, it may cause leaching or salt solubilization, and also may negatively affect the state of CH materials. To circumvent this problem, the so-called dry biocleaning approach has been devised, where dehydrated microbial cells could be employed without using free water or gel-based matrices. In this respect, the baker's yeast *Saccharomyces cerevisiae* has recently been successfully implemented on CH preservation [11]. Of note, dry biocleaning could be particularly efficient in removing alterations on highly ornamented stoneworks [11]. Still, the application of microorganisms – generally conceived as CH-deteriorating agents - continues to meet some reluctancy, which tends to favor the chemical and mechanical treatments [28]. Indeed, more work is needed to study the safety of biocleaning technology, in particular with respect to the CH objects themselves, but also regarding possible medical and environmental hazards [34]. Thus, the close monitorization of both short-term and long-term effects of bio-restored CH will help clarify whether this method is indeed an overall softer and safer process. This will also help decipher if microbial agents used for biocleaning need to be removed after usage at the CH site [17] or may remain under constant surveillance [34]. In any case, for the further use and acceptance of biocleaning strategies, it will be important to establish techniques and protocols to define if a given microorganism does cause biodeterioration, promotes only aesthetic alterations or, in fact, acts in a bioprotective manner [6].

Microbiology and CH cross paths at different levels: on the one hand, microbial activities fundamentally endanger CH, while, on the other hand, specific microorganisms can be employed to protect CH. In its Special Issue on CH, *Microbial Cell* has compiled and continues to gather a series of (research and review) articles that mirror this duality. The Special Issue further aims to serve as a platform for further scientific exchange in CH research: also for microbiologists, preserving the past is a cardinal task for a flourishing future.
